# Food (In)Security in Rapidly Urbanising, Low-Income Contexts

**DOI:** 10.3390/ijerph14121554

**Published:** 2017-12-11

**Authors:** Cecilia Tacoli

**Affiliations:** Human Settlements Group, International Institute for Environment and Development, 80-86 Grays Inn Road, London WC1X 8NH, UK; cecilia.tacoli@iied.org

**Keywords:** food security, urbanization, urban poverty

## Abstract

Urbanisation in low and middle-income nations presents both opportunities and immense challenges. As urban centres grow rapidly, inadequate housing and the lack of basic infrastructure and services affect a large and growing proportion of their population. There is also a growing body of evidence on urban poverty and its links with environmental hazards. There is, however, limited knowledge of how these challenges affect the ways in which poor urban residents gain access to food and secure healthy and nutritious diets. With some important exceptions, current discussions on food security continue to focus on production, with limited attention to consumption. Moreover, urban consumers are typically treated as a homogenous group and access to food markets is assumed to be sufficient. This paper describes how, for the urban poor in low and middle-income countries, food affordability and utilisation are shaped by the income and non-income dimensions of poverty that include the urban space.

## 1. Introduction

Debates on food security and nutrition have shifted in the past decade or so, with growing attention to consumption and especially to access, affordability and utilization. This is due to a number of interrelated factors that broadly reflect two transitions: the urban transition, with the majority of the world’s population now residing in urban centres [1], and the nutrition transition, with a growing proportion of overweight and obese individuals, often living in the same households as malnourished children [2]. Notwithstanding this shift in focus, food production remains to a large extent the predominant concern of policy-makers at global and national levels, as reflected in the Sustainable Development Goals, with little indication of an understanding of the major challenges in securing adequate diets facing the urban poor in low-income urbanizing regions [3,4].

Yet the evidence is clear. In the next three decades, it is expected that virtually all population growth will concentrate in urban areas of Africa and Asia [1]. Rapid urbanization will therefore take place mainly in regions where economic development and institutional capacity are generally weak. Hence, urban poverty is likely to become an increasingly pressing concern for national and local governments and for the global community. In this context, food security (or lack of it) will be a key element of poverty and one that is likely to stoke conflict if not addressed [5]. Urban food security is deeply interconnected with both income and non-income dimensions of poverty. For urban residents, access to food is primarily through market purchases, hence affordability and accessibility are key. Additionally, non-income factors such as inadequate infrastructure, housing and basic services, and exposure to environmental hazards are important if typically overlooked contributors to malnutrition and food insecurity. Space is increasingly recognized as central in the understanding of the challenges faced by the urban poor, from health issues [6] to gender relations [7].

This paper explores how a multiscale approach that includes the household, neighbourhood and city scales with particular attention to space, and specifically to urban slums, can provide an analytical lens that helps refine the understanding of the challenges faced by the urban poor in securing adequate diets. The importance of space, and of the non-income dimensions of poverty in urban areas, are briefly introduced in the next section. The paper then describes how disaggregating data on urban food security and nutrition uncovers deep inequalities and disturbing levels of ill-health among the urban poor. Section 4 and Section 5 describe respectively how food represents a very large proportion of the urban poor expenditure, and the equally important non-income dimensions of food insecurity and malnutrition. Section 6 describes the significant role that informal food retailers play in urban food systems of the poor. The concluding section considers how a multiscale approach to urban food security can help understand its complexity and develop better initiatives and policies.

## 2. Urbanization, Urban Space and Urban Poverty

Since about the first decade of this century it is estimated that more than half the world’s population live in urban centres. This is unprecedented, and it is also a relatively rapid transformation: only two centuries ago, this proportion was only about 5 percent of the world’s total population. Keeping in mind the rapid growth in the absolute size of the world’s population, the number of urban dwellers is also unprecedented. It is estimated that, based on current trends, by 2030 about 5 billion of the projected total of 8.1 billion people will live in urban areas [8]. 

Urbanization is a process that is deeply influenced by the scale and nature of economic, social and political change. This, in turn varies both between and within nations and regions, and helps explain why within a global process of urbanization there are often substantial local variations, including instances of de-urbanization that reflect economic decline or collapse, conflict and/or environmental disasters. So while there is a growing recognition of the economic benefits of urbanization, there is also a need to understand the context-specific factors that influence it [9]. This is especially important since economic growth alone does not necessarily result in inclusive urbanization, and in many instances entails risks of exclusion. 

In low and middle-income nations, urbanization is driven by net rural-urban migration responding to better economic opportunities in urban areas, or by the lack of opportunities in rural home areas. People’s movement reflects the spatial distribution of economic opportunities, and most of the economic growth in the past 60 years has been in urban centres. Today, around 97 percent of the world’s GDP is generated by industry and services, and around 65 percent of the world’s economically active population works in industry and services. Most of these activities are typically located in urban areas, where they can benefit from economies of scale and agglomeration economies [10].

In many low and middle-income nations, urban growth has been accompanied by the rapid expansion of unplanned, underserved neighbourhoods with high concentrations of poor people, or ‘slums’. The term comes with heavy intellectual baggage and has only recently re-entered the terminology and, as noted by some authors, is not necessarily here to stay [7]. Its implicit illegality has more often than not resulted in the stigmatization and marginalization of its residents, including evictions and the denial of access to basic services and rights. In the UN Habitat’s definition, slums consist of any households lacking one or more of the following conditions: access to improved water, access to improved sanitation, sufficient living space, durability of housing and secure tenure [11]. This definition highlights the diversity and complexity of slums, which challenges unified descriptions, but at the same time it lacks the spatial dimension which is crucial for infrastructure and basic services. It does however go beyond the much simpler definition of ‘informal settlements’ which in many instances can also apply to high income neighbourhoods in cities of the Global South. 

More recently, several authors have highlighted how slums are spatial entities with specific characteristics that are distinct from but contribute to the understanding of urban poverty and its implications for key issues such as health and gender relations [6,7]. Global interest in slums, reflected in their inclusion in the Sustainable Development Goals (Goal 11) and in the New Urban Agenda, is also linked to the growing number of urban residents living in these settlements: recent estimates suggest that 881 million people lived in slums in 2014, an increase from 689 million in 1990, and a figure that is projected to reach 2 billion by 2030 [8].

The lack of adequate, regular incomes is an important dimension of urban poverty as urban economies are essentially cash-based. There is also evidence that low-income consumers typically spend much more for goods and services of inferior quality than wealthier groups in what can be termed a ‘poverty penalty’. However, income is not the only dimension of urban poverty. Living in ‘slum’ settlements creates additional, non-income deprivations. These include the lack of policing, often resulting in high levels of violence and insecurity; lack of financial services and entitlement to vote, both of which usually require legal addresses and official land tenure documents; higher prices to purchase privately provided basic goods such as water and food, and services such as the use of latrines, school and health care—and also high costs of renting what is usually inadequate housing.

## 3. The Urbanization of Hunger and Malnutrition

The health outcomes of living in slum settlements are dramatic, and especially heavy for children—the still limited number of studies show that in many instances infant and child mortality rates can be higher than in rural areas, even when comparing slum children with rural children in the lowest socio-economic tercile [6]. Infectious diseases are also especially prevalent in crowded slums, and closely linked to lack of adequate water and sanitation, surface drainage and waste management [12,13]. Slum neighbourhoods have been identified as the most frequent source of cholera epidemics in African cities [6]. 

Not surprisingly, recurrent diarrhoea is a key reason for the prevalence of under-nutrition and stunted growth among slum children [14,15]. In many low and middle income nations, a very high proportion of children—in several instances up to one-third—are stunted or chronically malnourished. In 2005, over half the children in the poorest income quartile of India’s urban population were stunted, and an even higher proportion in two of India’s wealthiest states—Delhi and Maharashtra [16]. Such findings are especially disturbing because the deep inequalities that underpin them are likely to continue to affect future generations as chronic malnutrition lays the foundations for lifelong disadvantage, with challenges for cognitive development and future employment opportunities.

Malnutrition and stunting among children often goes hand in hand with increasing rates of overweight and obese adults in some of the most pernicious forms of nutrition transitions, and one that concentrates in urban slums. Research in Nairobi slums shows that 43 percent of overweight mothers and 37 percent of obese mothers have stunted children [17]. In Egypt, 16.2 percent of urban children suffer from stunting; at the same time, around 70 percent of women and almost half of men are overweight or obese [18]. 

Research in Bogota, Colombia, shows that while children in low-income areas have high levels of wasting (12.6 percent), almost 30 percent are overweight [19]. While this is a consequence of changes in lifestyle, with the reduction or disappearance of physical exercise, it is also the result of dietary changes and the growing reliance on food with high levels of fats and sugar and the decline in consumption of nutrient-rich food such as fresh fruit and vegetable, often unaffordable for the urban poor. Demographic and Health Survey data from seven sub-Saharan countries (Burkina Faso, Ghana, Kenya, Malawi, Niger and Tanzania) on the increase in urban overweight and obesity over a period of at least 10 years shows that the increase was highest among the poorest, at 50 percent, compared to the richest at only 7 percent [20].

## 4. Income Poverty and Food Insecurity

While urban agriculture can play a role in enhancing food security and dietary adequacy, its role should not be overemphasized as it is often quite limited, albeit with large inter-city variations [4,21,22]. Lack of sufficient and regular incomes is effectively the root cause of urban food insecurity. Urban residents rely primarily on food purchases, and any decline in incomes and/or increases in food prices can have catastrophic consequences. Research on how the food, fuel and financial shocks affected low-income groups in the period 2008–2011, showed food insecurity as the most severe cumulative impact [23]. A large majority of low-income urban residents rely on informal sector activities and casual labor that only provide low and irregular earnings. In low-income nations, it is estimated that informal employment accounts for half to three-quarters of all non-agricultural employment [24]. 

Food accounts for an extremely large proportion of low-income households’ total expenditure. Research in 11 southern African cities showed that, albeit with great variations between cities, food purchase is the most important expenditure for most households, and that it is greater among poorer households [25]. The same research suggests that four out of five poor urban households do not have enough to eat at any given time [26].

Research in one of Nairobi’s largest slum settlements, Mathare, suggests similar patterns. Food is the single largest expenditure for residents, accounting for nearly half of household expenses. The high rate of joblessness and low wages, and the unpredictable nature of casual labour within slum settlements, translate into generalized food insecurity for residents [27]. Moreover, in all but one of the neighbourhoods in the settlement, overall expenditure is regularly much higher than incomes, suggesting high levels of indebtedness. Any shock such as a sudden illness or loss of assets has devastating impacts on such stretched budgets. Similarly, in low-income areas of Colombo, Sri Lanka’s capital city and Kitwe in Zambia, 30 percent and 20 percent of households respectively report spending almost all their available income on food [19,28].

The higher proportion of income spent on food by low-income households reflects their limited financial resources. It also reflects the fact that there is a sometimes considerable difference in prices within cities and between different types of retailers. In Cape Town, such difference between supermarkets and small shops can be as high as 20–26 percent [29]. In Egypt, most poverty line studies take regional food price differences into account but miss significant intra-city differences. The residents of Greater Cairo’s ashwa’iyyat (informal/slum settlements) can pay much more for food than the residents of wealthier neighbourhoods. In part, this is because markets and supermarkets, where prices are lower, are not usually located near low-income settlements. For low-income households depending on daily wages, food has to be bought on a daily basis from local shops and street vendors in small quantities, and is typically much more expensive: for example, a 2 kg box of ghee costs 20 LE (Egyptian pound), while a small 80 gram pack costs 1 LE, or 20 percent more [18]. In Madurai, in the Indian state of Tamil Nadu, residents of low-income settlements who rely on daily wages can afford significantly lower quality of food and smaller quantities than their neighbours who earn weekly wages and can buy food in bulk. They also rely mainly on local shops for their daily purchases because, although prices are higher, most of them offer credit facilities [30]. 

To cope with high food prices and income insecurity, the urban poor use a number of strategies. The most frequent is reducing the quality and quantity of food consumed, including reducing dietary diversity, while at the same time reducing non-food expenditure including foregoing health care, and increasing work time [19,23,27,28,31,32,33]. Reduced calories intake combined with the need to work longer hours can have long-term detrimental consequences, including increased micronutrient deficiency disorders, higher incidence of disease, higher child and maternal mortality, poorer school performance and reduced worker productivity. It also disproportionately affects women, as they are often the last ones to eat and tend to forego food to ensure children have enough.

## 5. The Non-Income Dimensions of Urban Food Insecurity

Slum conditions add another layer to the nature of urban food insecurity. Lack of adequate housing and sufficient living space are considerable obstacles to buying food in bulk and at lower cost. In Greater Cairo, where poverty is assumed to be low and almost negligible compared to other Egyptian governorates, the majority of households in low-income settlements are tenants. To keep rental costs at a manageable level, housing is often overcrowded, with shared bathrooms and residents forced to cook in the same room where they sleep [18]. Storage of food is extremely difficult, and is exacerbated by lack of refrigeration facilities which are essential in Egypt’s hot climate. In Berta Gibi, a low-income community in Addis Ababa, all households are tenants living in dwelling units that belong to the state. Although rent is cheap, the 33 households share one latrine, one water tap and one kitchen. To bake injera, Ethiopia’s staple, women have to queue sometimes for several hours [34]. 

Inadequate access to water and sanitation facilities also have a detrimental impact on health and nutrition, as described earlier in this paper. Secure tenure also has important implications for food security. Access to social welfare benefits, including food rations, and eligibility to targeted poverty reduction programs require a legal address in the city, and access to financial services such as loans also require official land tenure documents. Securing these documents is all but impossible for the residents of slums and informal settlements [35]. 

Disproportionate exposure to environmental hazards is pervasive in slum settlements, which are in many cases built in typically unsuitable if not downright dangerous locations such as waste-contaminated land and land prone to flooding and/or landslides. This is the combined result of the need for the urban poor to be close to employment opportunities, and the limited availability of adequate land. These localized and everyday risks are exacerbated by the lack of basic infrastructure and services, exposing residents to water-borne, food-borne and vector-borne diseases. With climate change, the increased frequency and intensity of extreme weather events such as floods and heatwaves will also intensify health hazards and the loss of assets. These events also contribute to higher food prices as they affect different elements of food systems—from production to distribution and storage, retail and consumption. Inadequate diets in turn increase vulnerability to infectious disease while the higher incidence of ill-health contributes to malnutrition [36,37]. 

These impacts are also heavily gendered: women bear the primary responsibility for domestic and care work, and the lack or inadequate provision of basic services and unsafe environmental conditions so widespread in slum settlements disproportionately affect them. Engaging in paid work is in most cases a necessity for women in low income households, but long hours, often made worse by long journeys to work, and time-consuming reproductive activities—including care for the often sick children, preparing food in inadequate housing with limited if any dedicated cooking space [34]—take a toll in what is best described as women’s time poverty [38].

## 6. Informal Food Retailers

Perhaps unsurprisingly, purchasing cooked food from street vendors is a widely adopted strategy by the poorest urban groups whose incomes and living conditions make cooking their own food a challenge [30]. In Nigeria, urban residents spend up to half their food budget on street foods, while in Accra this accounts for 40 percent of low-income families’ food purchases [28]. In low-income settlements of Johannesburg, over 80 percent of households source food from informal vendors [39]. Consumption of street foods also tends to increase when food and cooking fuel costs rise since their price usually goes up more slowly as a result of economies of scale in production [28]. A similar growing reliance on purchases from vendors is documented in Mathare [40,41]. A systematic review of the nutritional contribution of street foods to the diet of people in low and middle-income nations found that they contribute significantly to the daily intake of protein, although it also raised concerns over the assumed high contribution of street food to fat, sugar, salt and carbohydrates intakes. Overall, from a public health perspective the review concludes that the use of street foods should be encouraged [42]. 

This positive assessment is not reflected in practice. Exclusionary practices aiming to remove street vendors from public spaces are pervasive in cities of low and middle-income nations. They range from large-scale violent evictions in cities such as Cairo, Harare and Luanda, to relocations to marginal locations with low pedestrian footfall and inadequate facilities, to ongoing harassment by corrupt officials demanding bribes [43]. In addition, street vendors operating within slum settlements are exposed to the same food safety hazards that their clients: limited storage facilities, inadequate water and sanitation infrastructure and lack of solid waste collection. Long distances from food markets and the cost of transport also constrain their ability to sell fresh food. Recognizing the specific role of street vendors and supporting them to improve the safety and quality of their products is a major opportunity for increasing the food security of the urban poor [41,43]. 

## 7. Conclusions

How we understand food security and nutrition of the urban poor largely determines initiatives and policies that aim to address the challenges they face. The evidence shows that these are considerable; it also shows that many of them are not directly related to food but rather to the specific characteristics of urban poverty. These, in turn, tend to be spatially concentrated in slum settlements. A multiscale analytical approach, overlapping with a spatial lens, can help identify the specific opportunities and challenges at each level, and from there provide pointers for possible action. 

At the household level, access to food is determined largely by affordability, that is, by incomes. Equally important, however, are the availability of time to purchase and prepare food and of space to cook and store food and the location of selling points. These non-monetary factors help explain the crucial and growing role of informal street vendors. Additionally, living in slums has important implications for health, and therefore for residents’ ability to engage in productive work. It also affects their rights and their ability to access initiatives that include nutrition programs. Finally, it dramatically increases exposure to environmental hazards that in turn affect food security and nutrition. 

These hazards and risks can also be understood at the neighbourhood level: lack of sanitation in crowded settlements clearly has impacts on the whole community. The lack of basic infrastructure also affects street vendors in several ways: food contamination is a constant concern, but so are violence and risk of theft. Hence a power black-out means that street vendors close down at dusk, leaving customers who work long hours and return home after dark with no way to buy what may well be the only meal of the day. When combined to environmental hazards such as floods, this has a cumulative, negative impact on local food security.

Local governments have an important role to play in urban food systems. This includes the provision of reliable transport infrastructure to ensure that perishable foodstuffs reach markets quickly and storage facilities help reduce waste. It includes urban planning such as the location and food markets and regulations affecting opening times. It also includes land management, as this is the first step to limit environmental hazards. However, local governments need support from national governments if they are to fulfil their responsibility to provide their populations with the basic services and infrastructure needed for urban living. Food-specific policies include giving adequate support to consumption and avoid focusing solely on production; and recognizing that ‘consolidated’ food systems that support switches to large private sector actors and supermarkets do not necessarily benefit the poor. However, perhaps what is most necessary is acknowledging that urban food insecurity and urban poverty need to be a central concern at all levels of governance, from the local to the global.
